# Krankheit und Sinn – einige philosophische Unterscheidungen

**DOI:** 10.1007/s00481-021-00659-6

**Published:** 2021-09-17

**Authors:** Ralf Stoecker

**Affiliations:** Abteilung Philosophie, Universität Bielefeld, 33501 Bielefeld, Deutschland

**Keywords:** Krankheitssinn, Sinn des Lebens, Meaning of disease, Meaning of life

## Abstract

Ist es sinnvoll, krank zu sein? – Diese seltsam anmutende Frage bietet den Auftakt für eine philosophische Untersuchung, was unter dem Sinn einer Krankheit verstanden werden kann und was das Interessante an diesen Konzeptionen ist. Ausgangspunkt ist die Feststellung, dass der Ausdruck „Sinn“ offenkundig mehrdeutig ist. Man kann damit sprachliche Bedeutung, Zwecke oder Motive meinen. Es kann aber auch um den narrativen Sinn im biographischen Leben oder um das Verhältnis der Krankheit zum Lebenssinn eines Menschen gehen. Gerade die letzten beiden Lesarten erweisen sich als aufschlussreich: die Einbindung der Krankheiten in den Lebenslauf und Krankheiten als Anstoß, sich über das eigene Leben Gedanken zu machen. Krankheiten könnten so den Effekt haben, ein sinnvolleres Leben zu führen, sie könnten uns aber auch die ultimative Sinnlosigkeit unseres Daseins vor Augen führen.

## Abstract (en)

**Definition of the problem:**

Is it meaningful to have a disease?—Apparently, this is an odd question. It is often assumed, however, that diseases can have meaning.

**Arguments:**

In the article differences in the meaning of the term in question (“Sinn”) are distinguished: linguistic meaning, purposes or motives, meaning in a narrative and contributions to the meaning of life. The last two interpretations, in particular, are of utmost importance: acceptance of disease as a part of life and disease as an impetus to reflect on the meaning of one’s life as a whole.

**Conclusions:**

In the latter sense, diseases might help us to lead a more meaningful life. However, diseases might also make it evident that ultimately there is absolutely no hope to lead a meaningful life.

*Ist es sinnvoll, krank zu sein? –* Das ist eine seltsame Frage. Derzeit leiden beispielsweise eine Vielzahl von Menschen an Covid-19 und die ganze Welt steht kopf, um zu verhindern, dass es noch mehr werden. Ist es trotzdem sinnvoll, sich mit Corona zu identifizieren? Natürlich nicht. Wie kann man dann aber behaupten, dass Krankheiten sinnvoll seien?! – Seltsam ist an dieser Frage aber nicht nur, dass sie anscheinend so leicht zu beantworten ist. Zu erkranken scheint auch gar nicht von der richtigen Art zu sein, um sinnvoll sein zu können. Sinnvoll sind Sachen, die wir tun, Krankheiten sind hingegen etwas, das mit uns geschieht.

Dessen ungeachtet gibt es eine lange Tradition, in der darüber nachgedacht wurde, welchen Sinn Krankheiten haben könnten. Was kann damit gemeint sein und was ist letztlich davon zu halten? Darum soll es in dem folgenden Beitrag gehen.

## Sinn als Bedeutung

Am Beginn steht die Feststellung, dass das Wort „Sinn“ selbst in unterschiedlichem Sinn gemeint kann. Damit ist seine erste Verwendung auch gleich schon genannt: Sinn als die semantische Bedeutung eines sprachlichen Ausdrucks. Dieser Sinn ist innerhalb einer Sprache konventionell festgelegt und erlaubt es den Sprecherinnen und Sprechern dieser Sprache, etwas auszudrücken, sich Nachrichten zukommen zu lassen und andere Sprechakte zu vollziehen, beispielsweise sich zu erkundigen, um etwas zu bitten oder sich zu warnen. In diesem semantischen Sinn haben Krankheiten aber klarerweise keinen Sinn. Sie zählen nicht zu unserer Sprache. Krank zu sein ist keine Form sich sprachlich zu äußern.

Allerdings haben wir nicht nur sprachliche Mittel zur Verfügung, um uns wechselseitig etwas mitzuteilen, uns zu warnen usw. In einer legendären Szene in Mario Putzos Film „Der Pate“ befördert die Mafia den abgeschnittenen Kopf eines Rennpferds unter die Bettdecke eines unbotsamen Filmregisseurs. Ein blutiger Pferdekopf ist kein sprachliches Zeichen, aber trotzdem ist es klar, was ihm die Mafia damit sagen wollte. Alles und jedes kann unter geeigneten Umständen als Zeichen dienen und in diesem Sinne einen Sinn haben. Warum nicht also auch eine Krankheit?!

Zeichen setzen allerdings einen Absender voraus. Jemandem etwas zu sagen ist eine Handlung. Der Pferdekopf ist ein Zeichen, weil die Mafia ihn so verstanden haben wollte. Krankheiten sind aber keine Handlungen. Kranke sind Patienten, nicht Agenten. Der Kranke wird nicht krank, um jemandem damit etwas mitzuteilen.

Vielleicht ist er aber der Adressat einer Nachricht. Dann müsste es einen anderen Absender geben. Wir reden zumindest manchmal so, als könnten Krankheiten Äußerungen unseres Körpers sein und daher ihren Sinn erhalten. „Was will dir dein Körper damit sagen, dass du heute schon wieder Rückenschmerzen hast?“ – Doch diese Redeweise ist offenkundig metaphorisch. Gemeint ist damit, dass die Rückenschmerzen ein Anzeichen, ein Symptom dafür sein könnten, dass mit dem Leben dieses Menschen etwas nicht in Ordnung ist, was sich dann in den körperlichen Beschwerden niederschlägt. Ein Körper (gegeben, dass er etwas anderes ist als der Mensch oder die Person selbst) hat keine Absichten, auch nicht die Absicht, einem Menschen etwas mitzuteilen. Botschaften stellen viel zu hohe Ansprüche an den Absender, als dass sie ein bloßer Körper erfüllen könnte.^1^

Damit Krankheiten Nachrichten, Zeichen sein können, braucht es also jemanden, der sie als Zeichen verwendet, aber weder der Patient selbst, noch sein Körper kommen dafür infrage. Was bleibt, ist die religiöse Vorstellung, Krankheiten könnten Botschaften Gottes sein. Das ist es, was beispielsweise in der hebräischen Bibel Hiobs Freund Elihu hinter dem quälenden Ausschlag vermutet, der Hiob befallen hat: „Auch warnt [Gott den Menschen] durch Schmerzen auf seinem Bett und durch heftigen Kampf in seinen Gliedern“ (Hiob 33.19).^2^ Der Ausschlag, so Elihu, war vielleicht eine Mahnung oder sogar eine Strafe Gottes.

Religiöse Krankheitsdeutungen spielen bis in die jüngste Zeit hinein eine große Rolle. Im Kontext der AIDS-Pandemie und auch im Rahmen der Corona-Pandemie sahen sich die christlichen Kirchen wiederholt mit der Frage konfrontiert, ob diese Krankheitsausbrüche als Strafen Gottes zu werten seien, was sie glücklicherweise energisch bestritten haben.^3^ Da es mir hier aber ausschließlich um ein säkulares Verständnis der Beziehung zwischen Krankheit und Sinn geht, werde ich dem im Folgenden nicht weiter nachgehen.

Auch aus säkularer Sicht ist aber ein Blick auf die äußere Rahmenhandlung der Hiob-Geschichte interessant. Sie beginnt damit, dass Satan Gott herausfordert. Hiob sei nur deshalb so fromm, weil er reich und gesund sei. Gott möchte das Gegenteil beweisen und plagt Hiob mit Unglück und Krankheit, um seine Standfestigkeit zu demonstrieren. Die Krankheit dient ihm also nicht (oder zumindest nicht nur) als Botschaft an Hiob, sondern vor allem als Beweis in seinem Streit mit Satan. Damit ist eine weitere, wichtige Bedeutung des Wortes „Sinn“ angesprochen, der Sinn als Zweck.

## Sinn als Zweck

„Es hat keinen Sinn, bei der Theaterkasse anzurufen, die Vorstellung ist schon lange ausverkauft.“ – „Sinn“ ist offenkundig häufig synonym mit „Zweck“. Hiobs Krankheit hatte den Zweck, den Teufel zu widerlegen. Es gibt aber auch profanere, säkulare Gründe, jemanden krank zu machen, beispielsweise in der biologischen Kriegführung oder bei medizinischen Menschenversuchen. Krankheiten haben hier den Zweck, den militärischen Feind zu schwächen bzw. zu neuen medizinischen Erkenntnissen zu gelangen. Doch dieser Sinn ist sicher nicht gemeint, wenn normalerweise nach dem Sinn einer Krankheit gesucht wird.

Ebenso wenig ist damit dasjenige gemeint, was manchmal als „Pathologisierung“ beschrieben wird: die Schaffung neuer Krankheitsentitäten zur Denunziation und Unterdrückung missliebiger Verhaltensweisen oder Einstellungen. Als die sowjetische Psychiatrie das Konzept der sogenannten „schleichenden Schizophrenie“ einführte, diente dies dem Ziel, abweichende politische Meinungen als Krankheiten abzustempeln, um Dissidenten daraufhin in psychiatrischen Krankenhäusern kalt stellen zu können.^4^ Doch abermals meint das heute niemand, der nach dem Sinn von Krankheiten fragt.

Anders als Nachrichten setzen Zwecke aber nicht immer einen Handelnden voraus. Es war eine wichtige Einsicht der Evolutionsbiologie, dass evolutionäre Prozesse eine Rechtfertigung für Mittel-Zweck-Beziehungen liefern können, ohne einen Schöpfer im Hintergrund annehmen zu müssen. Auch in einer durch und durch naturwissenschaftlich aufgeklärten Welt gibt es einen Platz für teleologische Tatsachen jenseits der Handlungen. Sie lassen sich als funktional im Rahmen der evolutionären Durchsetzung einer Spezies erklären. Und in diesem Sinn können Krankheiten zweifellos sinnvoll sein, insbesondere dann, wenn man auch Symptome wie Fieber, Schmerzen oder Panik als Krankheiten betrachtet. Sie haben einen evolutionären Nutzen für uns Menschen. Insofern haben sie einen evolutionären Sinn, aber das ist schwerlich schon der gesuchte Sinn einer Krankheit.

Die Feststellung, dass mit dem Sinn auch eine funktionale Rolle gemeint sein kann, öffnet nun eine weitere Deutung für die Frage nach dem Sinn von Krankheiten: ihre Funktion in einem größeren Ganzen, im sozialen Umfeld der oder des Kranken. Das setzt allerdings voraus, dass Krankheiten überhaupt eine solche Funktion innehaben, dass sie also funktional gesehen nicht nur Defekte oder Störungen darstellen, sondern einen Beitrag zum Funktionieren eines Systems leisten können. Ob man dies bejaht, hängt wesentlich davon ab, worin man dieses Funktionieren verortet. Jedenfalls ist es naheliegend, dass manche Krankheiten soziale Systeme stabilisieren können. Daran knüpfen beispielsweise system- und familientherapeutische Ansätze an, die darauf abzielen, das Patientenumfeld so zu verändern, dass Krankheiten ihre Funktion verlieren und ein entsprechender Heilungseffekt eintritt.^5^

Gerade die vage Grenze zwischen bloßer Funktion und dem intendierten Zweck zweckgerichteter Handlungen birgt aber die Gefahr, dass ein funktionaler Sinn schnell als gezielter Einsatz einer Krankheit aufgefasst wird und damit die Frage aufwirft, ob die Patientinnen und Patienten möglicherweise ihre Krankheiten selbst hervorgerufen hätten.^6^ Vielleicht ist das aber auch gar keine Gefahr, sondern deutet darauf hin, dass ich gleich zu Beginn meiner Untersuchung viel zu schnell davon ausgegangen bin, dass Krankheiten keine Handlungen der kranken Person selbst sein könnten. In einigen einflussreichen medizinischen Traditionen wird dies zumindest ausdrücklich angenommen.

## Sinn als Motiv

„Wir sehen, daß die Menschen erkranken, wenn ihnen infolge äußerer Hindernisse oder inneren Mangels an Anpassung die Befriedigung ihrer erotischen Bedürfnisse in der Realität versagt ist. Wir sehen, daß sie sich dann in die Krankheit flüchten, um mit ihrer Hilfe eine Ersatzbefriedigung für das Versagte zu finden.“ (Freud 1943, S. 52) – Sigmund Freud spricht hier nur über psychische Krankheiten, den weitergehenden Schritt zu den somatischen Krankheiten geht dann aber die Psychosomatik. So schreibt Viktor von Weizsäcker: „Aus dieser Biografie wird es meistens gelingen, der Frage ,Warum ist die Krankheit gerade jetzt aufgetreten?’ und der Frage ,Warum ist die Krankheit gerade hier aufgetreten?’, und somit dem Sinn dieser Krankheit und letztlich auch dieser selbst näherzukommen. […] Statt einen Konflikt oder ein Problem zu lösen, werden wir krank. Eine Flucht in die Krankheit also, ein Gewinn in der Krankheit“ (von Weizsäcker 1987, S. 369).

Hinter diesen Konzeptionen steht die Idee, dass unser begründetes Verhalten nicht allein aus absichtlichen Handlungen besteht, sondern dass es auch so etwas gibt wie unbewusste Absichten, Gründe, Motive, aus denen wir etwas tun, was sich dann auch nicht allein auf willentlich steuerbare Körperfunktionen beschränken muss. Von Weizsäcker illustriert dies an einem Beispiel: „Die Patientin brauchte die Reise, zu deren Antritt sie sich nicht entschließen konnte, die sie sich aber auch nicht abzusagen traute, nicht zu machen, da sie ja krank wurde […]“ (von Weizsäcker 1987, S. 369). Sie hat sich selbst krank gemacht und wurde so der Entscheidung enthoben.

Unter dem Sinn einer Krankheit kann also die Absicht verstanden werden, die die kranke Person unbewusst mit dieser Krankheit verbindet. Dabei stellt sich aus philosophischer Sicht natürlich die Frage, inwieweit die Annahme einer derartigen unbewussten Intention hinter der Krankheit haltbar ist. Zweifellos können Überlegungen, Entscheidungen etc. Einfluss auf den Körper ausüben, auch über das willkürliche Nervensystem hinaus. Der Entschluss z. B., eine lange geplante Reise abzusagen, kann einen ganz schön ins Schwitzen bringen. Außerdem sind wir uns längst nicht immer darüber im Klaren, aus welchen Gründen und Motiven heraus wir handeln. Es kann beispielsweise ein mühsamer Lernprozess sein, sich bewusst zu werden, dass es die Angst vor der Fremde und nicht die Sorge um den ökologischen Fußabdruck war, die einen zu der Absage einer Reise motiviert hat. Von diesen Selbstverständlichkeiten, denen jede philosophische Handlungstheorie und Philosophie des Geistes Rechnung tragen muss, ist es aber noch ein erheblicher Schritt zu dem Zugeständnis, dass es auch prinzipiell unbewusste Absichten hinter scheinbar unwillkürlichen Körperreaktionen geben könne, wie Freud und von Weizsäcker es annahmen. Wie groß dieser Schritt ist, hängt wiederum von den ontologischen *Commitments* ab, die man mit der Zuschreibung von Absichten, Wünschen und Motiven eingeht, führt also letztlich mittenhinein in das Leib-Seele-Problem.

Neben diesen ontologischen Schwierigkeiten ist die These, Krankheiten seien das Produkt unbewusster Motive der Kranken, aber auch aus moralphilosophischer Sicht fragwürdig. Sie macht die Patientinnen und Patienten tendenziell selbst dafür verantwortlich, erkrankt zu sein, und verweigert ihnen damit das Mitgefühl gegenüber ihrem widrigen Schicksal. Wer nicht verreist, weil sie das Pech gehabt hat, krank zu werden, verdient Empathie. Wer sich aber selbst reiseuntauglich gemacht hat, bekommt nicht nur deutlich weniger Mitleid, sondern muss sich auch vorhalten lassen, sich nicht einmal offen gegen die Reise entschieden zu haben. Der Umstand, dass sich die betreffende Person selbst ihrer verborgenen Motivation nicht bewusst war, lässt sie eher noch kümmerlicher dastehen.

Der erste Teil des Weizsäcker-Zitats oben erlaubt aber auch eine schwächere Lesart, die ohne die starke Annahme aufkommt, Kranke hätten ihre Krankheit letztlich selbst veranlasst: Die Frage, warum jemand gerade jetzt und hier krank geworden sei, kann auch darauf abzielen, die Krankheit in ein biographisches Narrativ einzubauen.

## Narrativer Sinn

Krankheiten bilden einen Teil unseres Lebens. Es ist aber nicht ganz einfach zu sagen, was das ist, ein Leben. Es ist sicher weniger als die Summe aller Dinge, die zu unseren Lebzeiten geschehen. Dass mein Nachbar heute heiratet, ist zunächst ein Teil seines Lebens, nicht meines. Andererseits gehört mehr zu meinem Leben als das, was unmittelbar mit mir geschieht. Und es gehört mehr dazu als dasjenige, was ich erlebe oder auch nur erfahre. Vielleicht kann man sagen: Es gehört alles dazu, was mich betrifft, etwas angeht, beeinflusst, womit ich mich beschäftige, was mich bekümmert, woran mir etwas liegt. Auf den ersten Blick klingt das viel zu vage und deshalb nicht besonders informativ. Beim näheren Hinsehen spricht es aber nur dafür, dass eben dies die Rolle der Rede vom eigenen Leben ist: Eine Erzählung zu liefern, die vieles, was sich zu den eigenen Lebzeiten ereignet hat, zusammenfasst und verbindet. Eine solche Erzählung müsste nicht notwendig das bloße Abbild einer Wirklichkeit (des Lebens dieses Menschen) sein. Sie könnte stattdessen ein essentieller Bestandteil unserer praktischen Vernunft und dadurch pragmatisch gerechtfertigt sein.

Das Grundgerüst dieser Narration wäre kausal, sie fügt zusammen, in was man involviert war, weil man in anderes involviert war. Andere Bestandteile der Geschichte wären sowohl Dinge, die einem zustoßen, wie Handlungen, die man begangen hat, äußere Ereignisse und innere, psychische Vorgänge. Darüber hinaus wäre die Geschichte immer wieder wertend: Man hat dieses oder jenes richtig oder falsch gemacht, es ist einem Gutes oder Schlechtes widerfahren, man hat Glück bzw. Pech gehabt. Und die Geschichte hätte eine Protagonistin oder einen Protagonisten, man selbst. Das eigene Leben wäre dann eine Geschichte, mit der wir uns identifizieren, identifizieren können oder vielleicht auch identifizieren sollen.^7^

Krankheiten können ganz unterschiedlich in eine solche Geschichte integriert werden. Erst einmal sind sie etwas, das den Kranken zustößt und das mehr oder minder starke Ursachen im bisherigen Leben und verschiedene Einflüsse auf den weiteren Lebensweg haben kann. Entsprechend können Krankheiten auch bewertet werden. Das Grippevirus, das man sich auf der Bahnfahrt eingefangen hat, ist eher Pech, die Sehnenscheidenentzündung hat man sich hingegen als leidenschaftlicher Gamer selbst zuzuschreiben. Und diese Wertungen können sich im größeren narrativen Kontext auch durchaus verändern. Krankheiten, die für sich gesehen leidvoll sind, können insgesamt gesehen etwas Gutes haben, angefangen von der ungeliebten Familienfeier, vor der einen die Grippe bewahrt hat, bis hin zum Entschluss, die eigene Spielsucht energisch in Angriff zu nehmen.

Dabei fällt die Geschichte, die das eigene Leben ausmacht, nicht vom Himmel. Sie ist das Produkt eigenen Überlegens und der Aushandlung mit dem sozialen Umfeld. Verschiedene therapeutische Ansätze bauen darauf, dass ein Prozess, in dem eine Patientin die Krankheit als Teil in ihre Lebensgeschichte einordnet, sie in ihrem Umgang mit der Krankheit stärken kann (*empowerment, recovery*).^8^ In der Literatur ist hier manchmal davon die Rede, Patientinnen und Patienten wollten nicht nur verstehen, *warum* sie erkrankt seien, sondern auch *wozu*.

Auf diese Weise nach dem Sinn einer Krankheit zu suchen, kann aber durchaus Schattenseiten haben. Erstens kann es auch entlastend sein, Krankheiten als extern anzusehen, als etwas, das einem von außen zustößt, ohne dass man es sich „anziehen“ müsste. Bei manchen Krankheiten gelingt dies offensichtlich leichter, gerade bei psychischen Störungen ist es aber ein schwieriges Thema. Ein psychoseerfahrener Patient hat es so ausgedrückt: „Den Profis gelingt es immer wieder, Menschen zu finden, für die ihre psychiatrischen Krisen etwas Gutes, ja sogar Sinnvolles sind. Das empfinde ich gelinde gesagt als äußerst provokant und auch irreführend. Wer sagt von einem gebrochenen Bein oder einer Lungenentzündung im Nachhinein, es sei sinnvoll gewesen?“ (Bock et al. 2014, S. 63).

Zweitens finden wir das Repertoire für die Integration von Krankheiten in unsere Lebensgeschichten (ebenso wie für die Integration aller anderen Episoden) in unserer öffentlichen Sprache, die wiederum durch eine lange Tradition des Krankheitsverständnisses geprägt ist.^9^ Bestimmte Krankheitsbegriffe sind deshalb reich an Konnotationen, die medizinisch längst überholt sind und aus denen wiederum nicht selten eine schiefe metaphorische Verwendung hervorgegangen ist. Krankheiten wie Krebs, Schizophrenie, TBC, AIDS sind in der Gesellschaft mit bestimmten Bildern von ihrer Entstehung, dem Charakter des kranken Menschen, der entsprechenden Lebensaussichten, eventuell auch der drohenden Gefahren verbunden, die sich leicht in die Konstruktion der eigenen Lebensgeschichte einschleichen. Das gilt natürlich vor allem dort, wo die betreffenden Krankheiten in der Öffentlichkeit stigmatisiert sind. Je stärker jemand versucht, eine als irgendwie verwerflich erachtete Krankheit als Teil in das eigene Lebens zu integrieren, umso größer ist die Last, sich der damit verknüpften Entwertungen zu erwehren. Die Philosophin Susan Sontag, die diesen Phänomenen nachgegangen ist, schreibt deshalb in ihrem Buch *Illness as Metaphor*: „Nothing is more punitive then to give a disease a meaning—that meaning being invariably a moralistic one“ (Sontag 1990, S. 59).

## Krankheit und Lebenssinn

Die Idee, dass wir uns das eigene Leben als eine Art Erzählung vorstellen sollten, lässt noch offen, woran sich diese Erzählung ausrichtet, was bestimmt, was man wie in sie integriert. Im therapeutischen Kontext ist aber nicht selten mitgedacht, dass es darum gehe, dem Leben insgesamt einen Sinn zu geben. Das wäre dann sozusagen der Fixpunkt der Erzählung, das „wozu“, an dem die einzelnen Episoden gemessen werden. Am deutlichsten findet sich diese Position bei Viktor Frankl, der davon ausgeht, dass der „Wille zum Sinn“ kennzeichnend für den Menschen im Unterschied zum Tier ist.^10^ Entsprechend sei es eine Aufgabe der Psychotherapie, Menschen bei dieser Suche Beistand zu leisten.

Damit wird ein Zusammenhang hergestellt zwischen der Frage nach dem Sinn von Krankheiten und der prominenten philosophischen Frage nach dem Sinn des Lebens.^11^ Frankl geht davon aus, dass die Beschäftigung mit dem Sinn unseres Lebens Einfluss auf die Genesung von Krankheiten haben kann, und die moderne Psychologie bestätigt diesem Zusammenhang. „Je mehr Sinnerfüllung ein Mensch erfährt, desto gesünder ist er – sowohl in seelischer wie auch in körperlicher Hinsicht“, schreibt die Psychologin Tatjana Schnell (2016, S. 114).^12^

Krankheiten können aber auch umgekehrt einen philosophischen Nutzen haben, durch den Anstoß, den sie geben können, sich mit der philosophischen Frage nach dem Sinn des Lebens zu beschäftigen. Wenn es überhaupt sinnvoll ist, von einem Sinn des Lebens zu sprechen, dann muss es möglich sein, mehr oder weniger sinnvoll zu leben. Allerdings ist es uns nicht immer bewusst, inwieweit wir tatsächlich sinnvoll leben oder nicht. Uns darüber Gedanken machen zu können, ist sicher eine wichtige menschliche Fähigkeit, nicht selten sind wir aber so in unser alltägliches Leben eingebunden, dass diese Reflexionsfähigkeit nicht zum Einsatz kommt. Wir leben in den Tag hinein, ohne uns darüber klar zu werden, wie sinnvoll das eigentlich ist, was wir tun.

Diese Überlegungen sind der Anknüpfungspunkt einer aktuellen Diskussion in der Philosophie zum Sinn des Lebens, die unter anderem von Susan Wolf initiiert worden ist.^13^ Wolf geht davon aus, dass es wichtigere und weniger wichtige Dinge gibt, in die wir unser Leben investieren können, und dass der Sinn unseres Lebens darin liege, unser Herz an etwas zu hängen, das es auch wirklich wert sei. Wolf nimmt damit ein Verständnis menschlicher Personalität auf, das ursprünglich von Harry Frankfurt geprägt wurde, demzufolge es einer Person wesentlich sei, so etwas wie ein eigenes Projekt zu haben oder zu entwickeln.^14^ Dabei ist aber nicht jedes Projekt gleich sinnvoll – Wolfs Position ist insofern teilweise objektivistisch –, also ist es wichtig für uns, den Sinn eines Projekts hinterfragen und reflektieren zu können. Im Prinzip, so Wolf, könne man das natürlich immer tun, aber es gibt eben besondere Gelegenheiten, bei denen wir eher dazu neigen, diese grundsätzlichen Überlegungen anzustellen. Das ist nun der Ort, an dem Krankheiten (vor allem unheilbare oder sogar tödliche Krankheiten) uns dabei helfen können, ein sinnvolles Leben zu führen. Sie können uns eine Auszeit geben, in der uns der Alltag nicht länger von der Reflexion abhält. Sie können uns dazu bringen festzustellen, was uns wirklich wichtig im Leben ist. Und sie können uns unsere Verletzlichkeit, Schwäche und Endlichkeit vor Augen führen. Der Sinn der Krankheiten ist hier ihr Wert für unsere Lebensgestaltung. Sie helfen uns, ein gutes, sinnvolles Leben zu führen.

## Krankheit und Sinnlosigkeit

Es gibt aber auch einen anderen, gegenläufigen Beitrag, den die Krankheiten für unsere Suche nach einem sinnvollen Leben leisten können, gerade auch durch die Schwäche und Vergänglichkeit, die sie uns vor Augen führen. Der römische Kaiser und Philosoph Marc Aurel erläutert dies mit zwei ernüchternden Gleichnissen: „Was hat ein Ball davon, wenn er in die Höhe geworfen wird, oder was passiert ihm Schlimmes, wenn er wieder herunterkommt und auf die Erde fällt? Was hat die Wasserblase davon, wenn sie entsteht, oder was passiert ihr Schlimmes, wenn sie sich wieder auflöst?“ (Marc Aurel 1998, S. 189). Wenn man krank wird, beginnt man vielleicht nicht nur zu grübeln, sondern gelangt dabei möglicherweise auch zu unerfreulich skeptischen Konklusionen. Führt uns der plötzliche Einbruch von Krankheit und Schwäche in unser Leben nicht gerade vor Augen, dass all unsere Pläne, Projekte und Perspektiven auf Sand gebaut sind, dass unser Auf und Ab im Leben wie das Steigen und Sinken eines Balls oder das Schwellen und Platzen einer Blase zu unserem Werden und Vergehen insgesamt gehört, das aber letztlich ebenso sinnlos und müßig ist wie alles andere auf der Welt – „eitel und Haschen nach Wind“ (Prediger 3.4)?!

In meinen Augen ist das der philosophisch spannendste Beitrag des Krankseins zum Sinn des Lebens. Natürlich ist es wichtig, darüber nachzudenken, wie man sein Leben insgesamt gestalten möchte. Krankheiten tragen dazu bei, erstens indem sie wichtige Episoden unseres Lebenslaufs darstellen können, die einen Platz in diesem Leben bekommen müssen, und zweitens, weil sie uns häufig den Anstoß dazu geben, uns Gedanken über unser Leben zu machen. Dies alles basiert aber auf der Grundüberzeugung, dass unsere Endlichkeit und die Unabsehbarkeit unseres Schicksals nicht zur Folge haben, dass all diese Bemühungen letztlich absurd sind, und es ist keineswegs klar, dass sich diese Grundüberzeugung halten lässt.^15^ Vielleicht braucht es erst einen radikalen Einbruch in unser vertrautes, vermeintlich sinnerfülltes Leben, wie es beispielsweisen eine Krankheit aber auch ein schmerzhafter Verlust oder ein unverhofftes Scheitern darstellen kann, um uns die Brüchigkeit dieses Anspruchs auf Sinn vor Augen zu führen.

## Fazit

Ist es sinnvoll krank zu sein? – Nein! Krankheiten sind etwas, was uns geschieht, nicht etwas, das wir uns zulegen, weil wir einen Sinn damit verbinden – auch keinen unbewussten. Aber die Tatsache, dass wir erkranken, bietet uns normalerweise einen Grund, uns über unser Leben Gedanken zu machen und darüber, wie es nach der Krankheit weitergehen sollte. Das ist zunächst sehr sinnvoll und möglicherweise kommt der Krankheit als einer Lebensepisode auch noch eine besondere Rolle darin zu, als besonders sinnvoller Teil des Lebens. Nicht wenige Menschen, die in den letzten Monaten eine Covid-19-Erkrankung durchgemacht haben, sehen, wie sie es selbst beschreiben, die Welt heute mit anderen Augen.

Ob unsere Lebensgeschichte allerdings tatsächlich eine solche Pointe haben kann, einen Lebenssinn, auf den wir das Leben beruhigt ausrichten können, das hängt davon ab, ob sich unser Leben nicht letztlich doch als sinnlos erweist und alle Suche nach dem Sinn deshalb vergeblich ist. Covid-19 hat uns nicht nur unsere Vergänglichkeit vor Augen geführt, sondern auch gezeigt, wie ausgeliefert wir immer noch einem Schicksal sind, in dem Glück und Pech eng beieinanderliegen. Wo jemand den Skiurlaub verbracht hat, ob er ein Hotel oder eine Apotheke betreibt, ob die Krankheit still oder katastrophal verläuft, das bekommt plötzlich existentielle Bedeutung. Ist es wirklich sinnvoll, in einer derart gefährlichen, unberechenbaren Welt, in der am Ende immer der eigene Tod steht, ein sinnvolles Leben führen zu wollen? Krankheiten sind ein guter Anlass, sich auch darüber Gedanken zu machen.^16^

## Footnotes

1 Es ist zudem durchaus fraglich, wie sinnvoll es außerhalb eines ontologischen Leib-Seele-Dualismus ist, zwischen einem Menschen und seinem Körper zu unterscheiden (vgl. Stoecker 2010, Kapitel 3).

2 Vgl. Bieberstein (2009).

3 Vgl. Gelsner (1987) sowie die Meldung in den Vatican News vom 30.03.2020: „‚Corona ist keine Strafe Gottes‘ – Die beiden großen Kirchen in Deutschland wenden sich übereinstimmend gegen die These, das Coronavirus als Strafe Gottes zu sehen“ (https://www.vaticannews.va/de/kirche/news/2020-03/corona-virus-kirche-strafe-gott-wilmer-bedford-bibelwerk-deutsch.html. Zugegriffen: 21. April 2021).

4 vgl. van Voren (2010).

5 Vgl. Schweitzer et al. (2007).

6 Vgl. Schweitzer et al. (2007, S. 8).

7 Die freizügige Bezugnahme auf Narrationen ist philosophisch allerdings nicht unproblematisch. Vgl. dazu die kritische Diskussion in von Wedelstaedt (2016).

8 Vgl. die Beiträge in Bock et al. (2014).

9 Dass dies auch tröstlich sein kann, führt Johannes Fischer (2011) überzeugend aus.

10 Vgl. Frankl (2015) und dazu: Kipke (2018).

11 Ein anderer Zusammenhang zwischen Krankheit und Lebenssinn besteht insofern, als sich die Bezeichnung „Krankheit“ auch auf eine bestimmte Haltung zu dieser Sinnsuche beziehen kann, etwa bei William James, der den Pessimismus als „religiöse Krankheit“ bezeichnet (James 2000), oder bei Sören Kierkegaard, für den jeder Mensch, der noch nicht zum wahren Christentum gefunden hat, in einer existentiellen Verzweiflung als einer „Krankheit zum Tode“ lebt (Kierkegaard 1976). Krank ist aus dieser Sicht, wer den (religiösen) Sinn im Leben noch nicht gefunden hat. Mit dieser Verwendung werde ich mich im Weiteren nicht beschäftigen.

12 Vgl. hierzu auch den Ansatz der *narrative medicine, *z. B. in Greenhalgh (2009).

13 Kipke (2014); Vgl. Wolf (2000).

14 Vgl. Frankfurt (1999).

15 Ein sehr empfehlenswerter Aufsatz zu diesem Thema ist Nagel (1984).

16 Ich möchte Roland Kipke, Jens Kulenkampff und Katharina Woellert sowie den beiden anonymen Gutachtenden der „Ethik in der Medizin“ für hilfreiche Informationen und Anmerkungen danken.
